# Histological and chemical diagnosis of a combat lesion in *Triceratops*

**DOI:** 10.1038/s41598-022-08033-2

**Published:** 2022-04-07

**Authors:** Ruggero D’Anastasio, Jacopo Cilli, Flavio Bacchia, Federico Fanti, Giacomo Gobbo, Luigi Capasso

**Affiliations:** 1 Department of Medicine and Aging Science, ‘G. D’Annunzio’ University of Chieti–Pescara, Chieti, Italy; 2 Zoic Limited Liability Company, Trieste, Italy; 3 Department of Biological, Geological, and Environmental Sciences, Alma Mater Studiorum University of Bologna, Bologna, Italy

**Keywords:** Evolution, Zoology

## Abstract

In the collective imagination derived from scientific and popular literature, *Triceratops* often faced each other in combat. Thus, from the second half of the twentieth century, these ceratopsids were described as pugnacious animals. This arises primarily from the interpretation of extracranial fenestrae in ceratopsids being the result of combat trauma. However, the diagnosis of the traumatic nature of these anatomical variants of their neck frill requires evidence of bone healing and remodelling by microscopy analysis. Here, we present the case of the *Triceratops horridus* known as Big John, which is one of the largest specimens discovered in the Hell Creek Formation (Upper Cretaceous; South Dakota, USA). Its right squamosal bone shows an extrafenestra with irregular margins and signs of inflammation. Microscopy analysis revealed newly formed and healing bone, with histological signs typical of the bone remodelling phase. Chemical analysis revealed sulphur that was derived from glycosaminoglycan’s and sulphated glycoproteins of the preosseous osteoid substance present in the healing phases of a bone trauma. Histological and microanalytical analyses confirm that the squamosal fenestra of Big John is the result of a traumatic event, which might indeed have occurred during combat with another *Triceratops*.

## Introduction

The Late Cretaceous dinosaur *Triceratops* was characterised by its large neck frill, which predominantly consisted of the hyperelongated parietal and squamosal bones. It also had two large supraorbital horns and a smaller nasal horn. This cranial ornamentation of *Triceratops* probably served several functions. The evolutionary predecessors of ceratopsids had a relatively developed frill, while the horns, if present, were reduced to simple protuberances. The primitive function of the frill, therefore, was that of visual display and/or species recognition^1^.

The development of the solid frill and the long supraorbital horns during the evolution of ceratopsids such as *Triceratops* suggests that their function was to protect the cervical region of the skull from blows inflicted by individuals of the same species. Farke et al. stated that in *Triceratops* the horns not only had a visual function, but were also used in intraspecific combat^2,3^. Therefore, the cranial ornaments in *Triceratops* were used not only as a display, but also as a means of attack and defence against conspecifics^3^.

**Figure 1:**
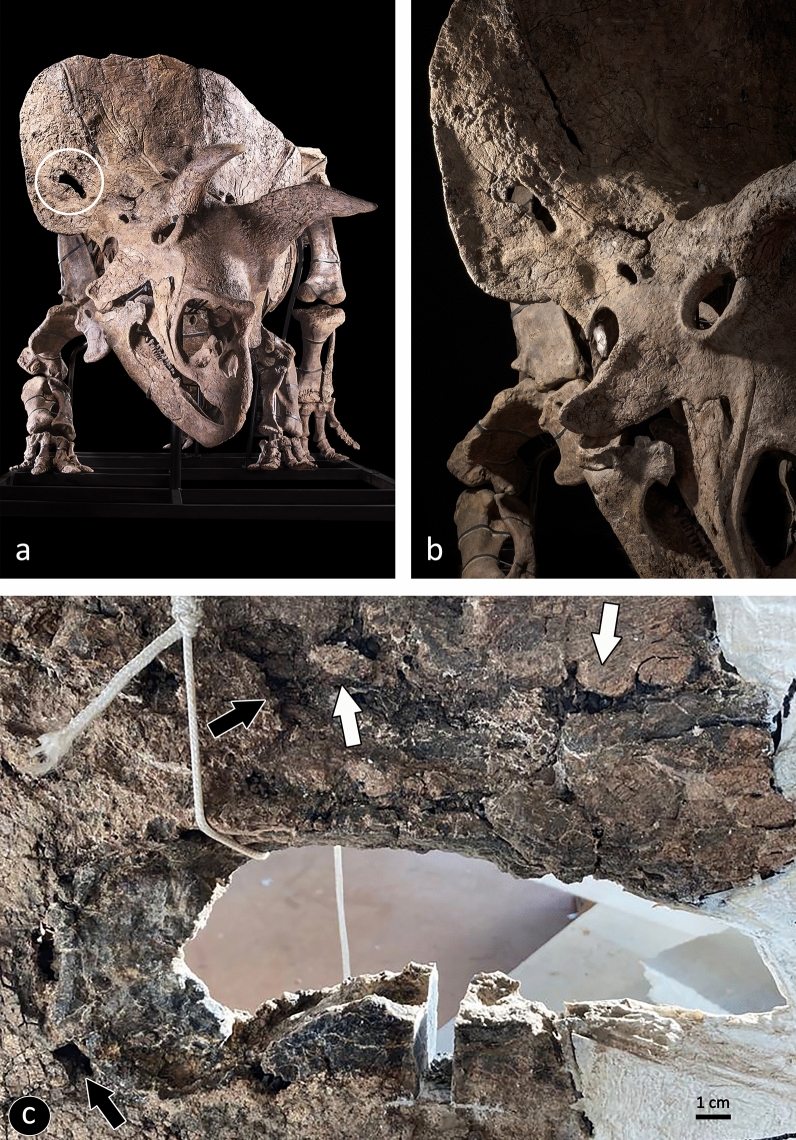
The *Triceratops horridus* Big John. (**a**) The complete restored skeleton; the fenestra analysed here is indicated by the white circle (courtesy of Ferrara A., and Briano I.). (**b**) Detail of the skull (courtesy of Ferrara A., and Briano I.). (**c**) Detail of the fenestra: plaquelike deposition of reactive bone (white arrows) and lytic lesions (black arrows) are visible on the bony surface around the lesion. The region where the sample to be analysed was taken is visible on the lower margin.

The specimen of *Triceratops horridus* known as Big John (due to its large size) was discovered in 2014 in the Upper Cretaceous Hell Creek Formation (South Dakota, USA), from where numerous remains of Ceratopsidae (Chasmosaurinae) have been recovered^4–6^ (see Supplementary Information). Big John has a fenestra on the right squamosal that completely perforates the bone (Fig. 1a,b). The presence of squamosal fenestrae has been documented for the chasmosaurines, which are usually unilateral and with variable shapes, from oval to circular. Their aetiology stimulated a debate that generated two hypotheses. The first hypothesis claims that squamosal fenestrae are of traumatic origin, probably as the result of intraspecific fighting or attacks by predators, such as *Tyrannosaurus*^7,8^. The second hypothesis argues that the squamosal fenestrae are not pathological, but are the result of bone resorption related to aging, or of bone removal for bone that was no longer necessary from a biomechanical and structural point of view^9^.

Macroscopic evidence of associated traumatic injuries, such as bone calli, radial fracture lines and displacement of bone fragments, should contribute to our understanding of whether these fenestrae are indeed traumatic lesions or are the outcome of other pathologies (e.g., infectious diseases). Histological analysis of the affected areas is a valuable tool for ascertaining the pathological nature of such lesions, to potentially exclude the fenestrae as taphonomic lesions or an anatomical variant linked to aging.

In modern mammals and reptiles, post-traumatic bone remodelling involves recruitment of osteogenic cells and activation of the molecular and chemical mechanisms that control the formation of soft calli that are rich in procollagen and proteoglycans, and their subsequent calcification^10–14^. The composition and distribution of the biochemical elements in the healing bone also changes during the healing process^14–17^. Consequently, the study of these factors can also provide useful information for diagnosis of the possible traumatic nature of these lesions.

### Macroscopic and microscopic analyses of the squamosal fenestra of Big John

The fenestra on the right squamosal bone of Big John is shaped like a ‘keyhole’, with the widest part positioned caudally. The rostrocaudal length of the squamosal bone is 112 cm; the distance of the caudal border of the fenestra from the caudal apex of the squamosal is 75 cm; its rostrocaudal diameter and mediolateral width are 20 cm and 5 cm, respectively.

The lumen of this fenestra is partially filled with a ring of brownish material, which is particularly evident on the caudal and inferior edges (Fig. 1c). The bone surface around the fenestra is irregular and characterised by plaquelike depositions of reactive bone that could resulted from periostitis; i.e., a non-specific inflammatory process, which can be triggered by different stimuli (e.g., trauma, infection)^18^.

**Figure 2:**
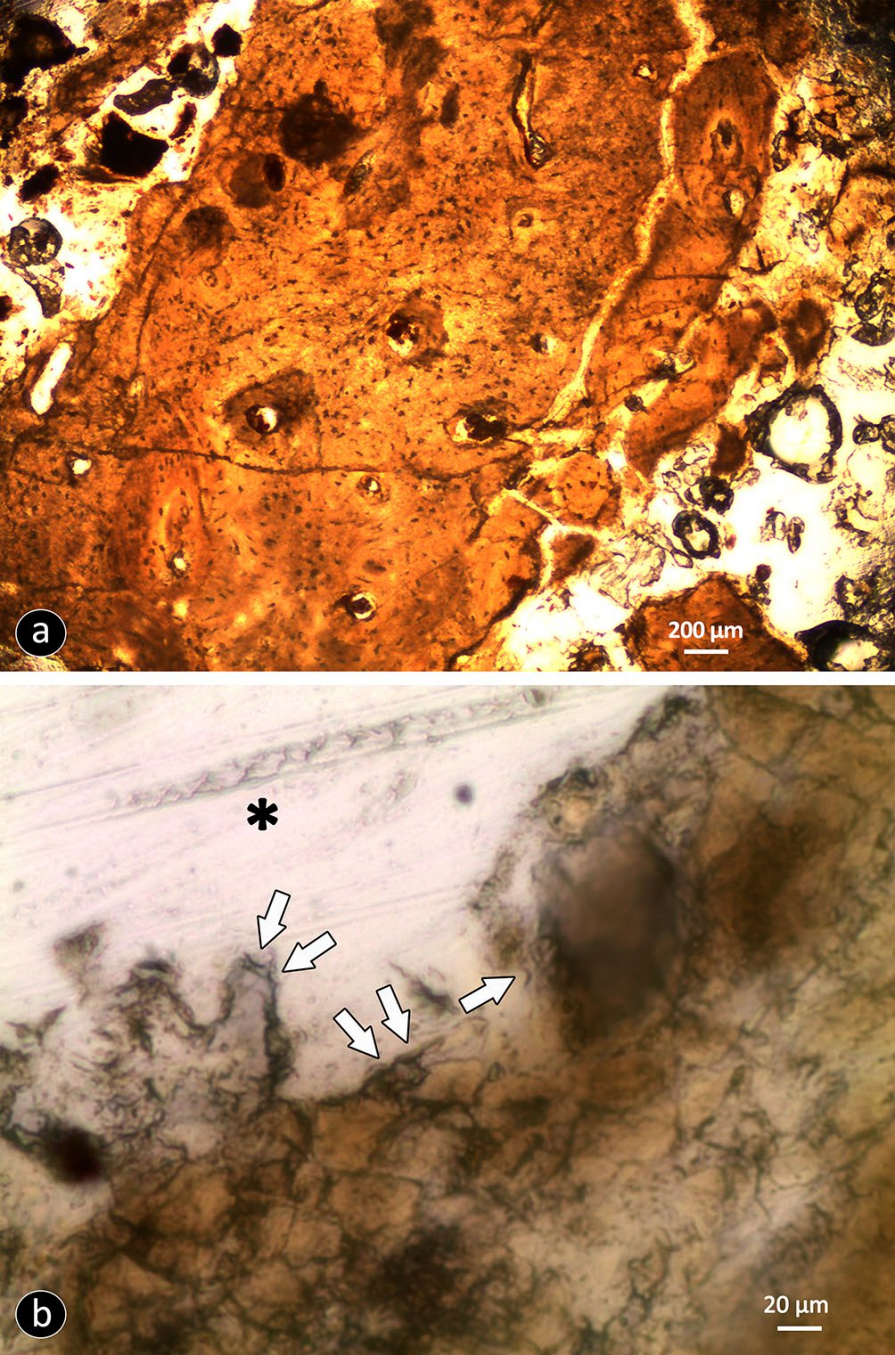
Fragment of the bone tissue that delimits the fenestra of Big John. (**a**) The bone is well vascularised, and osteocytic lacunae are visible. (**b**) Small areas of bone resorption are visible along the cortical margin: these areas have regular shapes and dimensions that correspond to Howship lacunae (white arrows; the asterisk indicates the lumen of the lesion).

**Figure 3:**
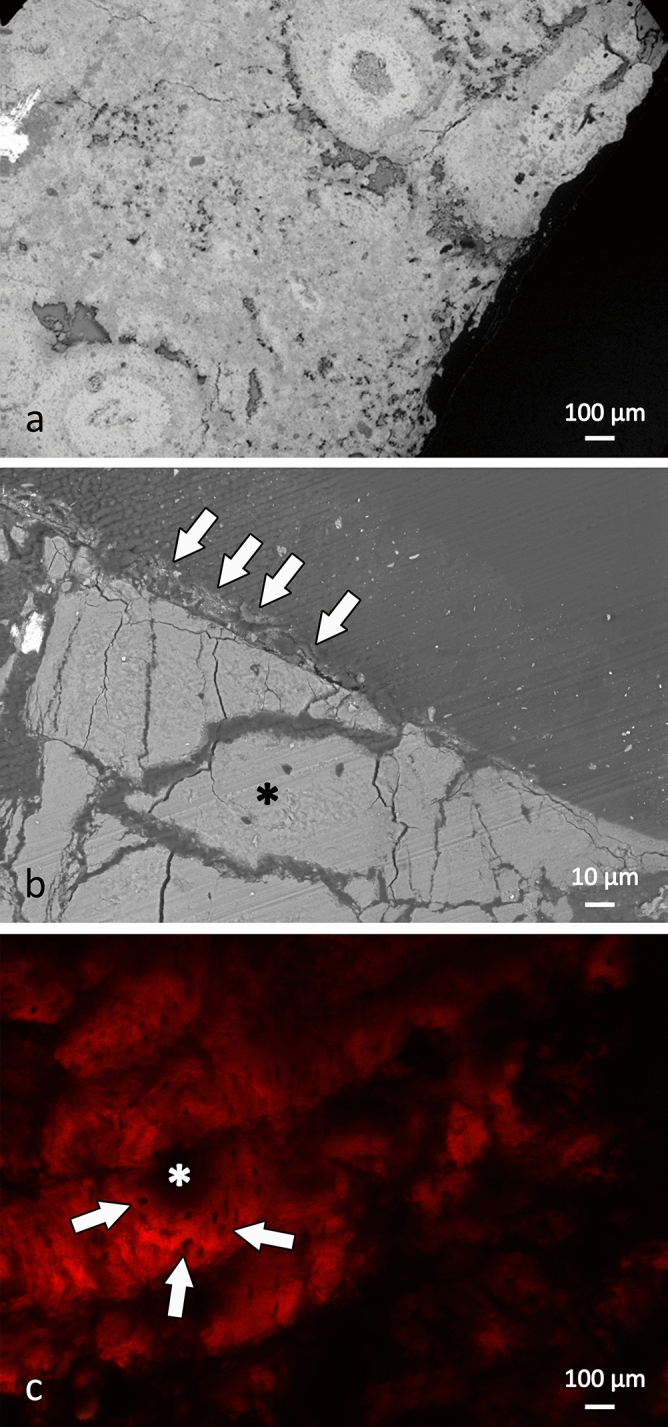
Optical and scanning electron microscopy (SEM) analysis. (**a**) Bone tissue at the margin of the lesion: it is porotic and well vascularized (SEM). (**b**) The interphase between normal and healing bone: the white arrows show a layer of bone matrix on the edge of the lesion that was undergoing mineralisation (SEM). Bone tissue in the region furthest from the lesion is compact and poorly vascularized (black asterisk). (**c**) The bone tissue delimiting the lesion: haversian canals (asterisk) are surrounded by osteocytic lacunae (arrows) (transmitted light optical microscopy).

The histological analyses of a sample taken from the lower margin of the fenestra (Supplementary Fig. S1) provided information on the characteristics of the bone tissue near and distant from the edge of the squamosal opening. The optical and scanning electron miroscopy (SEM) images show that the bone tissue that delimits the fenestra was well vascularised, with primary osteons and osteocytic lacunae visible (Figs. 2, 3). At high magnification (× 400), the border shows areas of bone resorption, the morphology and dimensions of which are compatible with Howship lacunae, which are seen as eroded depressions of the bone surface due to the activity of osteoclasts (Howship lacunae mean diameter, 19.38 ± 2.25 μm) (Fig. 2b). Conversely, the bone tissue distant from the fenestra is characterised by its compact structure, with little vascularisation.

**Figure 4:**
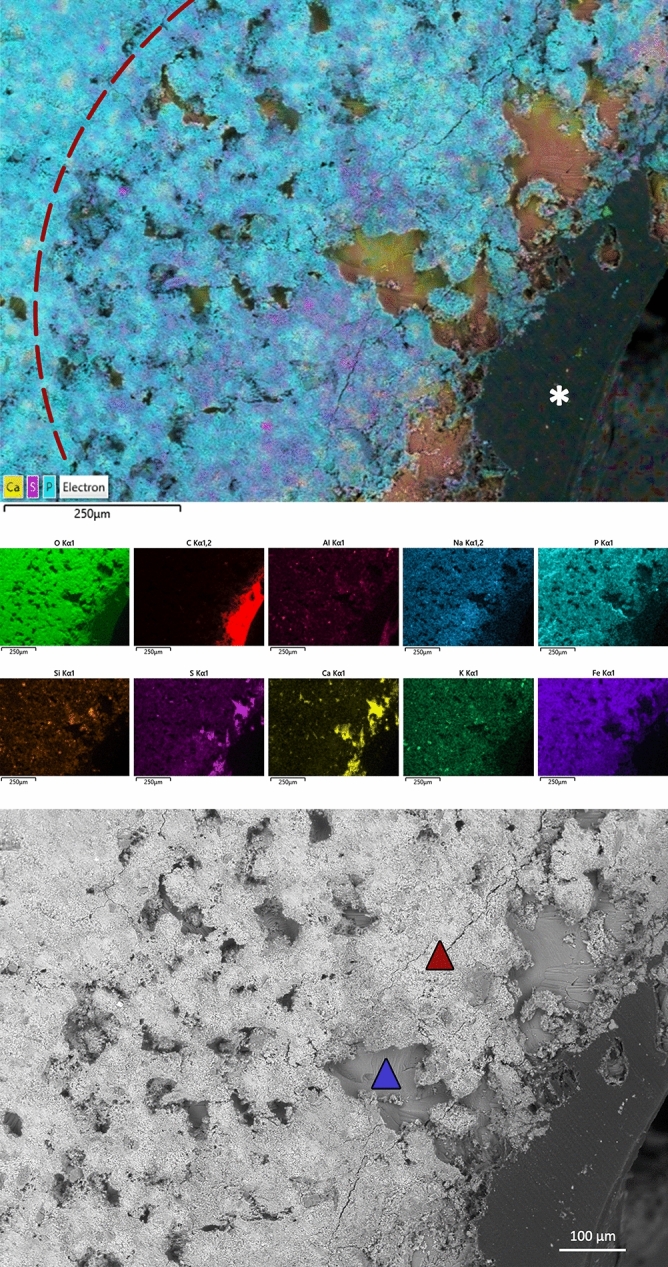
Distribution map from electronic scanning field emission microscopy for the chemical elements in the bony region that delimits the lesion. (**a**) The main image shows the calcification front of the osteoid substance, where mineralised bone (blue; phosphorus) has advanced towards the edge of the lesion, to replace the osteoid substance (yellow; calcium), which is rich in glycosaminoglycan’s and sulphur (violet). Below: distribution maps of each chemical element (Oxford Aztec Live Microanalysis system with detector Ultim Max 100, version 5.0; EDS OXFORD) (the dash line: calcification front; asterisk: the lumen of the lesion). (**b**) Magnification of the borders between mineralised bone (red triangle) and osteoid substance (blue triangle) (SEM).

The dominant chemical elements in the bone sample seen under the electronic scanning field emission microscope equipped with a microanalysis system were oxygen (O), calcium (Ca) and phosphorus (P) (Supplementary Figs. S2, S3). The other elements seen were iron (Fe), sulphur (S), potassium (K), sodium (Na) and aluminium (Al), in smaller quantities and variously distributed, as well as traces of barium (Ba), strontium (Sr) and manganese (Mn). By focusing the microanalysis on the margin of the lesion, the presence of S was noted in the area that delimits the fenestra, with a distribution superimposable on that of Ca. The S is almost completely absent in the bone tissue distant from the lesion, where P, O and Na are the predominant elements (Fig. 4).

## Discussion

Macroscopy analysis of the bone surface that delimits the fenestra on the right squamosal bone of Big John show alterations that could be consequent to inflammatory processes^18^. In anatomical region where the periosteum is close to the skin surface, localized ossifying periostitis, sometimes accompanied by a lytic response, can be observed secondary to trauma^12^.

Histological and chemical analyses of the bone sample from the margin of the fenestra demonstrate that it consists of newly formed bone tissue that appears porous and disorganised, with numerous vascular canals and osteocytic lacunae (Figs. 2a, 3a), where layers of bone matrix that were undergoing mineralisation tend to fill the opening (Fig. 3b). At higher magnification, the margin of the bone tissue facing the lumen of the fenestra shows resorption areas, with morphology and dimensions that correspond to the Howship lacunae that are characteristic of the remodelling of human bone tissue (Fig. 2b)^19^. All of these histological characteristics are compatible with previously metabolically active and remodelling bone.

The specific cellular and molecular processes that occur during bone remodelling in modern reptiles and dinosaurs are not known in detail. Therefore, the reference interpretative models here are the physiology and biochemistry of healing bone tissue in mammals (including humans). Bone remodelling begins with a phase of bone resorption by osteoclasts. Through a combination of acid dissolution of the mineralised matrix and digestion of the organic matrix by proteolytic enzymes, small areas of bone resorption are formed (the so-called Howship lacunae); these are concave in shape and a few microns in diameter^18,19^. The bone resorption is followed by a phase of apposition of osteoid substance by the osteoblasts, which undergoes mineralisation through precipitation of calcium phosphate.

The main constituents of the mineralised bone matrix in new bone are Ca, O and P (which are typically present in calcium hydroxyapatite), and also S (Fig. 4). The phases of bone resorption and neoformation are separated by a transition phase, during which macrophage-like mononuclear cells deposit an initial layer of bone matrix that forms the cementing line that delimits the osteons^20^. This cementing line is rich in sulphated proteins^21^, which explains the presence of S among the elements that constitute bone during the mineralisation phase.

The distribution of S in repaired bone tissue might also be linked to glycosaminoglycan’s and glycoproteins in the osteoid substance, which is the preosseous substance found in ossification centres. Glycosaminoglycan’s contain sulphate and are essential for osteogenesis^22–24^. Baylink et al. demonstrated that the concentration of glycosaminoglycan’s and S are elevated in the osteoid substance, and then are reduced during the mineralisation process, as a result of enzymatic digestion^10^. The results of the studies carried out by Baylink were confirmed by Takagi et al., who showed a reduction in S in areas undergoing mineralisation and in the calcified matrix^25^. The removal of glycosaminoglycan’s and their sulphate compounds might be the prerequisite to the start of the calcification process of the osteoid substance. The SEM images in the present study showed the mineralisation front of the bone tissue in the remodelling phase, whereby the distribution map of the elements highlighted the presence of S in the areas that had still been occupied by the osteoid substance that had not yet calcified (Fig. 4).

The bone that was more distant from the margins of the lesion was mainly made up of bone matrix with few vascular channels and reduced numbers of osteocytic lacunae. These are histological characteristics that are compatible with compact bone tissue that has completed or almost completed the remodelling phase. In this region, moreover, there were lower amounts of S, which confirms the hypothesis of a gradual reduction in S during deposition of hydroxyapatite in the osteoid substance.

The edge of the lesion thus appeared to be made up of remodelling bone tissue undergoing mineralisation, as seen for both the light and dark areas of the histological preparations. Conversely, the innermost region that was more distant from the fenestra showed well mineralised bone or bone that was in an advanced state of mineralisation.

The results of the histological and chemical analyses show that the bone that circumscribed and partially filled the lesion had been made up of metabolically active and remodelling tissue (Figs. 2, 4). The fenestra is therefore of traumatic origin, and at the time of the death of Big John, the lesion was still healing.

The presence of newly formed bone and the histological characteristics of bone remodelling that was in progress excludes the possibilities that the lesion occurred post mortem (i.e., that it is of a taphonomic nature) and that the fenestra represented an anatomical variant. This conclusion is supported by the lack of regular, sharp and blunt margins for the lesion. Furthermore, although congenital squamosal fenestrae have been described for the subfamily of *Chamsmosaurinae*, they have not been found in *Triceratops* or *Anchiceratops* to date^9^. Instead, the lesion was possibly caused by the horn of another *Triceratops*. The mediolateral diameter (equal to 5 cm) and shape of the caudal region of the fenestra coincide with those of the apex of the supraorbital horns. Furthermore, the *Triceratops* used their horns in intraspecific combat^3^.

Farke hypothesised three models of engagement in the intraspecific combat of *Triceratops*^2^. The “single horn contact position” defines the contact that occurs between only one postorbital horn of each combatant, where lesions of the squamosal bone are inflicted by the nasal horn. In the “oblique horn locking position”, the skulls come into contact when they are slightly rotated with respect to each other, and contact occurs between both postorbital horns of both individuals. This model predicts lesions inflicted by the nasal horn in the rostral portion of both the parietals and squamosal’s. The third model of engagement is defined as “full horn locking”, which consists of the collision between the skulls when tilted at about 45° with respect to the horizontal plane, and involves contact between both postorbital horns of the opponents. This combat mode will result in injuries in the region of the temporal fenestra. The location of the lesion analysed here on the squamosal bone of Big John does not respect these predictive models of Farke for horn use in *Triceratops*^2^. It appears likely that the wound was instead inflicted from behind Big John (Supplementary Fig. S4), whereby the rival’s horn would have penetrated the frill and then slipped towards the rostrum, giving this lesion the shape of a keyhole. According to Aufderheide and Rodrìguez-Martìn^26^ pointed weapons could generate a perforating lesion with sharply-defined edges. The blow may also have caused comminution and loss of fragments and radiating fissures, that were hidden by bone remodelling in the months following the trauma. In describing his models on the trauma inflicted by the horns in the fighting between *Triceratops*, Farke did not exclude that there might also be other engagement dynamics besides those he hypothesised^2^.

Big John appears to have survived this trauma for some time. Two cases have been described in the literature where adult specimens of *Centrosaurus* survived extensive traumatic lesions to the skull^9^. However, it remains difficult to establish how long Big John survived following this trauma. Although the bone remodelling process can be first recognized in the third to fourth weeks after the trauma^11^, the healing times vary according to the species and to the extent of the trauma. Considering the healing times of traumatic injuries in modern reptiles, along with the size of the traumatic injury and the amount of bone repair, it is likely that the death of Big John occurred at least 6 months after this traumatic event^27,28^.

This study confirms the existence of intraspecific fighting in *Triceratops*. Furthermore, although the physiological and cellular mechanisms underlying the healing process in dinosaurs are still largely unknown, it would appear to be similar to those described in humans and mammals. Further histological and microanalytical investigations on fossil remains with traumatic lesions might shed light on the bone physiopathology of these reptiles.

## Materials and methods

The histological sample of *Triceratops horridus* known as Big John is housed at Museum of the University “G. D’Annunzio” (Chieti, Italy) (access number 23751). The microscopy and histological analyses were performed on a bone sample taken from the inferior margin of the fenestra (Supplementary Data Fig. S1). The sample was analysed under stereo microscopy (Wild M8; Leica). The 30-μm-thick histological sections were made using a diamond-edged circular blade microtome (RMS-16G3; Reha-Tech Engineering). It was not possible to make thinner sections as the fossilized bone tissue tended to fracture and fragment excessively. The sample was cut directly from the edge of the fenestra, without any pre-treatment. The histological sections were analysed under a transmitted light optical microscopy (BX41; Olympus). The observations were conducted under white and polarised light. Both the sample and the histological sections were examined under electronic scanning field emission microscope (GeminiSEM 500; Zeiss) equipped with a microanalysis system (Oxford Aztec Live Microanalysis system, version 5.0; EDS OXFORD) and a detector (Ultim Max 100). The analyses were carried out in ‘analytical’ mode (15 kV; working distance, 8.5 mm; variable pressure); this allowed analysis of the samples directly, without any preliminary treatments with glues or surface metallisation.

## Supplementary Information

Supplementary Information.
