# Author Correction: Histological and chemical diagnosis of a combat lesion in *Triceratops*

**DOI:** 10.1038/s41598-022-11359-6

**Published:** 2022-04-28

**Authors:** Ruggero D’Anastasio, Jacopo Cilli, Flavio Bacchia, Federico Fanti, Giacomo Gobbo, Luigi Capasso

**Affiliations:** 1 Department of Medicine and Aging Science, ‘G. D’Annunzio’ University of Chieti–Pescara, Chieti, Italy; 2 Zoic Limited Liability Company, Trieste, Italy; 3 Department of Biological, Geological, and Environmental Sciences, Alma Mater Studiorum University of Bologna, Bologna, Italy

Correction to: *Scientific Reports* 10.1038/s41598-022-08033-2, published online 07 April 2022

The original version of this Article contained errors, where the place of discovery of *Triceratops horridus,* known as Big John, was incorrectly stated as ‘Hell Creek Formation (Upper Cretaceous; MT, USA).’

As a result, in the Abstract,

“Here, we present the case of the *Triceratops horridus* known as Big John, which is one of the largest specimens discovered in the Hell Creek Formation (Upper Cretaceous; MT, USA).”

now reads:

“Here, we present the case of the *Triceratops horridus* known as Big John, which is one of the largest specimens discovered in the Hell Creek Formation (Upper Cretaceous; South Dakota, USA).”

Additionally, in the third paragraph of the Introduction,

“The specimen of *Triceratops horridus* known as Big John (due to its large size) was discovered in 2014 in the Upper Cretaceous Hell Creek Formation (MT, USA), from where numerous remains of Ceratopsidae (Chasmosaurinae) have been recovered^4–6^ (see Supplementary Information).”

now reads:

“The specimen of *Triceratops horridus* known as Big John (due to its large size) was discovered in 2014 in the Upper Cretaceous Hell Creek Formation (South Dakota, USA), from where numerous remains of Ceratopsidae (Chasmosaurinae) have been recovered^4–6^ (see Supplementary Information).”

The original Article has been corrected.

