# Supplementary material for: Histological and chemical diagnosis of a combat lesion in Triceratops
Source: Sci Rep. 2022 Apr 7;12:3941. doi: 10.1038/s41598-022-08033-2 (PMC8990019; doi:10.1038/s41598-022-08033-2)
Supplement: Supplementary file 1 — Supplementary Information. [file 41598_2022_8033_MOESM1_ESM.pdf]

Supplementary Information

Histological and chemical diagnosis of a combat lesion in *Triceratops*

Ruggero D'Anastasio[1]*, Jacopo Cilli[1], Flavio Bacchia[2], Federico Fanti[3], Giacomo Gobbo[3], Luigi Capasso[1]

[1]Department of Medicine and Aging Science, 'G. D'Annunzio' University of Chieti–Pescara, Chieti, Italy

[2]Zoic Limited Liability Company, Trieste, Italy

[3]Department of Biologial, Geological, and Environmental Sciences, Alma Mater Studiorum University of Bologna, Italy

Geology and Taphonomy

A partial skeleton of a large Triceratops nicknamed 'Big John' has been restored and mounted between 2020 and 2021 in Trieste (Italy), under the supervision of Zoic. The specimen is a partial, disarticulated skeleton of a mature individual collected between 2014 and 2015 in South Dakota (U.S.A.). The main quarry was discovered in 2014 in the Hell Creek Formation at the Mud Butte Ranch, Southwestern Perkins County, 36 miles NE of Newell, South Dakota. GPS coordinates for the site are 45° 03' 11.26" N, 102° 52' 50.582 W. The exact stratigraphic occurrence of the site remains uncertain as no measurable section is exposed in the proximity of the site. However, it is here considered as representative of the upper Hell Creek Formation, thus Maastrichtian (latest Cretaceous), on the light of the taxonomic assignment of 'Big John' to *Triceratops horridus* based on cranial features including differences in relative length of the postorbital horn cores (long in *T. horridus* and shorter in *T. prorsus*), morphology of the rostrum (elongate in *T. horridus* and shorter in *T. prorsus*[29,30,31,32,33]. Mapped elements indicate pre-burial disarticulation of the individual, although their distribution and preservation suggest no major transportation or re-arrangements due to physical factors (hydraulic reworking). Overall, more than 200 identifiable elements were

prepared and consolidated. For this study, ribs and frill elements were sampled for histological analyses to investigate age and pathologies of this individual. Samples are currently housed in the Museum of the University "G. D'Annunzio" (Chieti, Italy) under the access number 23751.

A reference quarry map and detailed list of preserved elements are available upon request.

References

29. Longrich, N. & Field, D. Torosaurus is not Triceratops: ontogeny in Chasmosaurine ceratopsids as a case study in dinosaur taxonomy. *PLoS ONE* **7**, e32623 (2012).

30. Maiorino, L., Farke, A., Kotsakis, T. & Piras, P. Is Torosaurus Triceratops? Geometric Morphometric Evidence of Late Maastrichtian Ceratopsid Dinosaurs. *PLoS ONE* **8**, e81608 (2013).

31. Horner, J. & Goodwin, M. Major cranial changes during Triceratops ontogeny. *Proc. R. Soc. B* **273**, 2757–2761 (2006).

32. Scannella, J. & Horner, J. Torosaurus Marsh, 1891, is Triceratops Marsh, 1889 (Ceratopsidae: Chasmosaurinae): synonymy through ontogeny. *J. Vertebr. Paleontol.* **30**, 1157- 1168 (2010).

33. Scannella, J., Fowler, D., Goodwin, M. & Horner, J. Evolutionary trends in Triceratops from the Hell Creek Formation, Montana. *Proc. Natl. Acad. Sci. USA* **111**, 10245-10250 (2014).

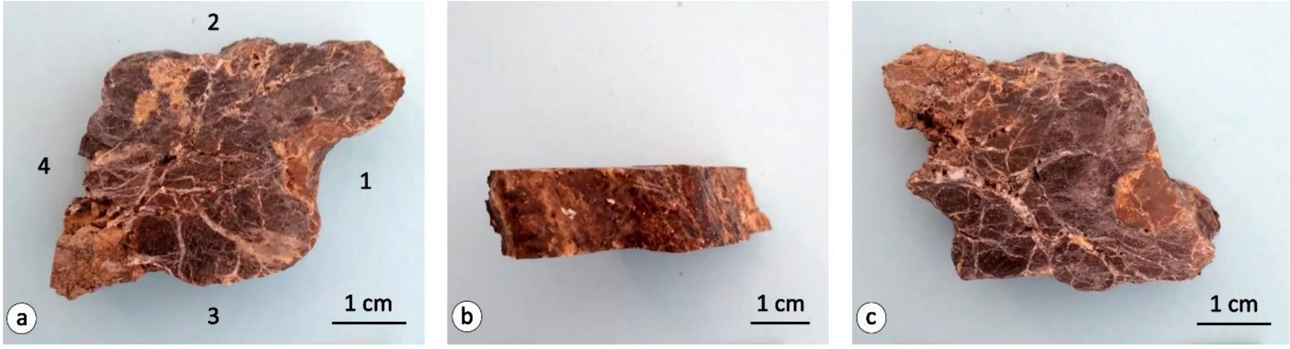

Supplementary Figure S1. The bone sample from the squamosal bone of Big John (maximum length, 43.19 mm; maximum height, 30.49 mm; maximum width, 11.59 mm) (access number 23751). (**a**) Distal surface. 1, superior border; 2, distal border; 3, anterior border; 4, inferior border. (**b**) Superior view. (**c**) Anterior surface. The different colouring of some areas of the sample is linked to crystalline formations of different chemical compositions.

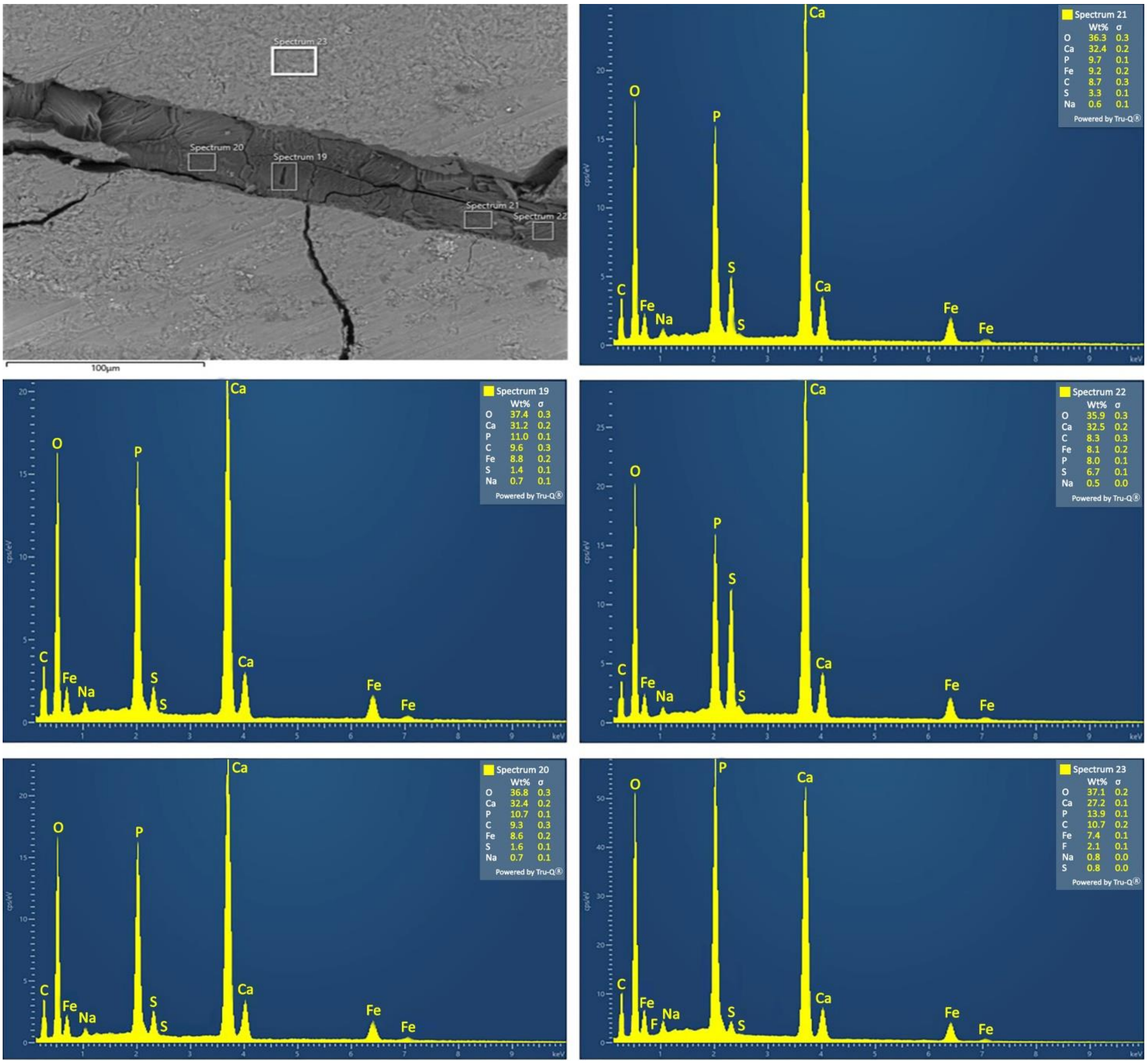

Supplementary Figure S2. Microanalysis of the bone sample from Big John. The elements most present are oxygen, phosphorus and calcium, the constituent elements of calcium hydroxyapatite, the main component of the mineralised bone matrix. The squares in the first panel represent the areas where the chemical analyzes (spectra) were carried out (Oxford Aztec Live Microanalysis system with detector Ultim Max 100, version 5.0; EDS OXFORD).

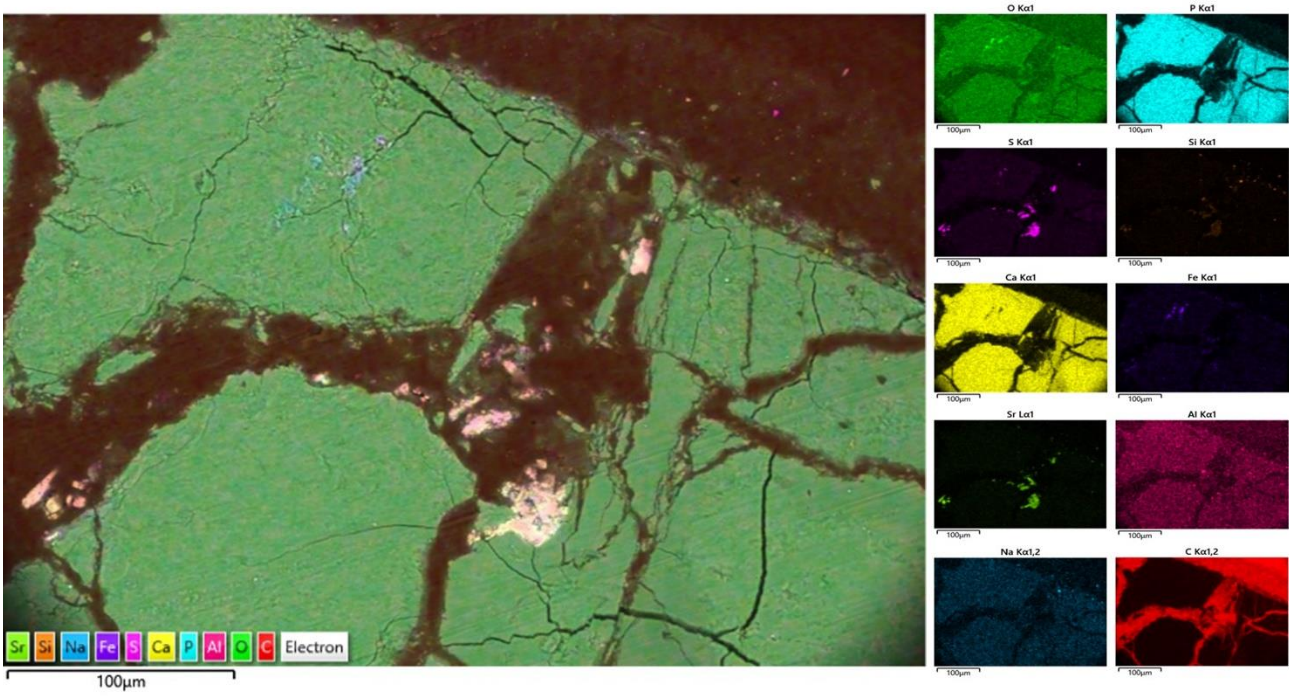

Supplementary Figure S3. Distribution map of the chemical elements in the bony region that delimits the lesion. The thin layer at the edge of the lesion comprises phosphorus (blue; P), calcium (yellow; Ca) and oxygen (green; O), the elements that characterise the bone matrix. Right: distribution maps of each chemical element (Oxford Aztec Live Microanalysis system with detector Ultim Max 100, version 5.0; EDS OXFORD).

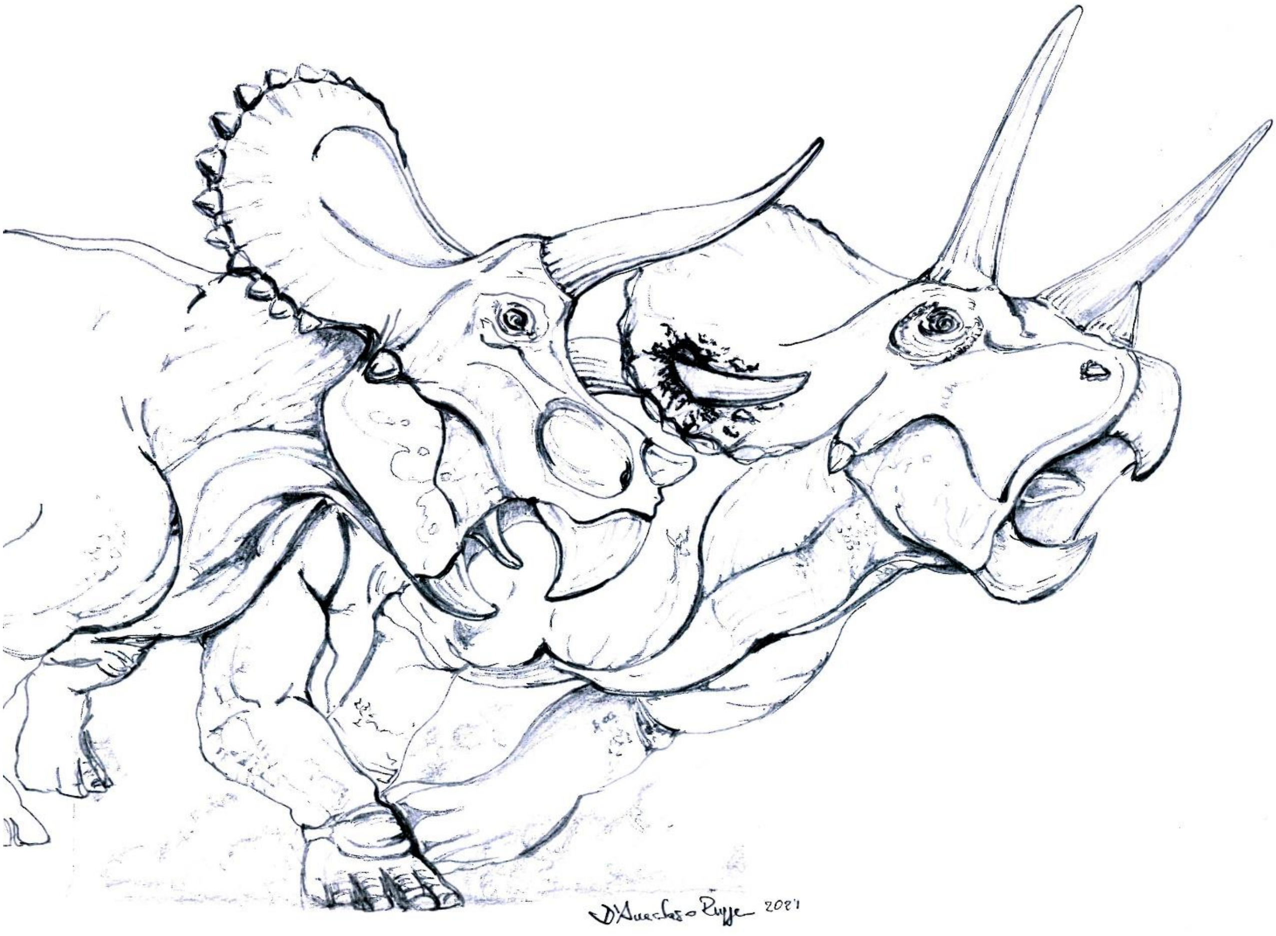

Supplementary Figure S4. Representation of the possible dynamics of the fight between Big John and another triceratops (©Ruggero D’Anastasio).
